# 

**Published:** 1896-04

**Authors:** 


### CONTENTS.

ORIGINAL ARTICLES:
- Milk as a Factor in the Causation of Disease. By JOHN M. PARKER, D.V.S. . . . 253
- Osteoporosis. By GEORGE C. FAVILLE, D.V.M. . . . . . . . . 258
- Bone-softening. By M. D. HOGE, JR., M.D. . . . . . . . . 262
- Heredity. By W. T. RUSSELL, V.S. . . . . . . . . . . 265
- A Non-classified Equine Disease in Montana. By ROBERT H. BIRD, M.R.C.V.S. 270
- Glanders in Horses. By DR. E. H. BIART . . . . . . . . . 274
- Actinomycosis. By J. L. WINGATE . . . . . . . . . . 276
- Tuberculosis. By DR. JOHN FORBES . . . . . . . . . . 281

ABSTRACTS FROM FOREIGN JOURNALS:
- Swine-plague in Austria—Rib-fistula in a Cow—Acute Primary Peritonitis in Cattle—Sterilization of Tuberculous Meat—Belgian Law against Tuberculosis in Cattle—Filaria in the Eye of a Dog—Fern-extract in Tapeworm—Atresia Ani et Recti—Result of Abortion on Milk—Parenchymatous Mastitis Causing Disease in Humans 286–293

REPORTS OF CASES:
- Some Interesting Canine Cases. By CECIL FRENCH, D.V.S. . . . . . 294
- Aspiration-pneumonia Following Abscess of Steno's Duct. By JOHN GREER, JR. 296
- Embolism of the Aorta. By DR. OTTO NOACK . . . . . . . . 298
- A Cow with Chronic Cough that Did Not React. By L. O. LUSSON, V.M.D. . 299
- Traumatic Ventral Hernia . . . . . . . . . . . 301
- A Misplaced Molar. By W. J. MARTIN, V.S. . . . . . . . 302
- Fracture of the Trapezium. By JOHN WENDE, V.S. . . . . . . 302

CORRESPONDENCE . . . . . . . . . . . . . . . . 304

EDITORIAL—Our April Number—The Spoils-system—Another Unsuccessful Effort—Legislation in New York State—The Profession's Interests Above the Individual 306–313

LEGISLATION—New York State Veterinary Examining Board—New Jersey—Pennsylvania—Ohio—District of Columbia—National—Manitoba . . . . . 315–323

CONTROL WORK—New York, Pennsylvania, Maine, Massachusetts, Missouri, New Jersey, Delaware, Vermont, Great Britain . . . . . . . . . . 313

SOCIETY PROCEEDINGS—U. S. V. M. A.—North Dakota—Massachusetts—Montreal—New York County—Keystone—Schuylkill Valley—Wisconsin—Ontario College—Pennsylvania—McGill University, Comparative Psychology Society . 329–347

COMMENCEMENT EXERCISES—Indiana, Kansas City, McKillip, American, and New York Veterinary Colleges . . . . . . . . . . . 347–348

THERE IS ALWAYS SOMETHING NEW—NEW INVENTIONS—PERSONAL . . 348–356

